# How many genetic options for evolving insecticide resistance in heliothine and spodopteran pests?

**DOI:** 10.1002/ps.3542

**Published:** 2013-06-20

**Authors:** John G Oakeshott, Claire A Farnsworth, Peter D East, Colin Scott, Yangchun Han, Yidong Wu, Robyn J Russell

**Affiliations:** a CSIRO Ecosystem SciencesCanberra, Australia; b School of Biological Sciences, Australian National UniversityCanberra, Australia; c Cotton Catchment Communities CRCNarrabri, Australia; d College of Plant Protection, Nanjing Agricultural University, Key Laboratory of Monitoring and Management of Crop Diseases and Pest Insects (Ministry of Agriculture)Nanjing, People’s Republic of China

**Keywords:** Helicoverpa, Heliothis, Spodoptera, organophosphates, pyrethroids, genomics

## Abstract

The widely accepted paradigm for the development of insecticide resistance in field populations of insects is of selection for one or a very few genes of major effect. Limited genetic mapping data for organophosphate and pyrethroid resistance in heliothine and spodopteran pests generally agrees with this paradigm. However, other biochemical and transcriptomic data suggest a more complex set of changes in multiple P450 and esterase gene/enzyme systems in resistant strains of these species. We discuss possible explanations for this paradox, including the likely embedding of these genes in regulatory cascades and emerging evidence for their arrangement in large clusters of closely related genes. We conclude that there could indeed be an unusually large number of genetic options for evolving resistance in these species. © 2013 Society of Chemical Industry

## 1 INTRODUCTION: CONVENTIONAL WISDOMS ON THE GENETICS OF INSECTICIDE RESISTANCE

The dominant paradigms which emerged from several decades of classical genetic work on insecticide resistance were that major gene inheritance accounts for high level resistance to insecticides in the field and polygenic inheritance explains the generally lower level tolerance generated in selection experiments in the laboratory.^1^ The rationale for the field results was that only genes conferring high level resistance will be useful against the high doses of insecticide to which field populations are exposed, but that the large sizes of field populations means that such genes can be recovered even if they are initially very rare, or even absent until new mutations occur. By contrast, laboratory selection experiments almost always use lower insecticide doses that can select for various genes of smaller effect because the much smaller sizes of laboratory populations means that only relatively common, pre-existing mutations are likely to be captured and higher insecticide doses could kill the whole population. Notably most of the data from which these paradigms emerged involve resistances/tolerances to the older organophosphate (OP) and organochlorine (OC) chemistries in various Diptera (both flies and mosquitoes).

Subsequent molecular work has generally borne out these paradigms and, at least for target site resistance in the field, revealed a marked tendency for the same or very similar mutations to be found in orthologous systems in different species. Evidence for the latter phenomenon now encompasses resistance to carbamates and pyrethroids, as well as the OPs and OCs.^2^–^4^ There is also one case of metabolic resistance to OPs where one or other of the same two amino acid substitutions in orthologous carboxylesterases in several species confers significant OP hydrolase activity on the enzyme and resistance on the insect.^5^,^6^ Most of the molecular data involve Diptera again, as well as some Hemiptera (certain aphids and plant hoppers), but there is also mounting evidence that the parallelism of the resistance mutations extends to some other insect orders, and even into some non-insect groups such as Acari.^7^ One overall conclusion has therefore been that only a very finite number of genetic options are available through which field populations of insects can evolve insecticide resistance.

This conclusion has also been borne out by experiments involving laboratory mutagenesis and selection for high level resistance in *Drosophila*. Unlike the laboratory selection experiments that do not use mutagenesis above, these experiments are suited to the recovery of major gene effects. Work with several of the chemistries above plus the newer neonicotinoids and spinosyn has recurrently recovered the same resistance mutations in independent experiments, and these mutations are similar, and sometimes identical, to those found in field populations.^4^

## 2 EMERGING PATTERNS AND PARADOXES

At the same time, however, evidence is accumulating for certain species, particularly lepidopterans (but also including OC resistant houseflies^8^), suggesting that much more complex genetics may apply to some cases of field resistance. Large numbers of studies on several major pest lepidopterans such as the heliothines and spodopterans have correlated resistance with both simply inherited target site mutation and more complex changes in a range of putative detoxification enzymes [cytochrome P450s, esterases and glutathione *S*-transferases (GSTs)].^9^–^11^ Moreover, in cases such as the esterases, which are amenable to isozyme analysis, several different gene–enzyme systems have been implicated in the metabolic resistance.^12^

Some of the findings from the application of modern genomic technologies to insects from a variety of orders are also reinforcing this notion that many genes could potentially be involved in field resistance. Whole genome sequencing is revealing large numbers of genes in the P450, esterase and GST families that could be candidates for detoxification roles for the chemical insecticides; figures of ∼50–130, ∼20–70 and ∼10–40, respectively, are commonly found for these families in the insect genomes so far characterised. Notably, it appears that clades within these families associated with dietary and detoxification functions may contain many more members in polyphagous pest species in which resistance has recurrently emerged than in more host-specialised species less prone to develop resistance.^13^–^15^ Several of the Lepidoptera for which the genetics of resistance appear to be most complex are highly polyphagous pests.^10^,^11^

The emerging use of transcriptomics is also indicating higher levels of expression of several P450s, esterases and GSTs in insecticide resistant strains. Moreover, this pattern is evident even in several of the Diptera. In particular, an ‘*Aedes* Detox Chip’ containing over 200 P450, esterase and GST genes has been used in a series of microarray studies (validated with quantitative rtPCR work) that have implicated higher levels of expression of multiple P450 genes as well as several esterase and GST genes in OP and pyrethroid resistance in various field and laboratory-selected populations of *Ae. aegypti*.^16^–^18^ While these associations do not themselves establish causal connections between the elevated expression levels and detoxification/resistance, some aspects of the data, such as correlations between the expression levels and the levels of OP resistance during the course of selection and then the relaxation of that selection in the laboratory,^16^ certainly reinforce the idea. Interestingly also, the number of the detoxification genes implicated in the pyrethroid resistance appears to be inversely correlated with the frequency of target site resistance across different populations.^17^

‘Next gen’ genome resequencing technologies have not yet been deployed for genome-wide mapping of quantitative trait loci (QTL) contributing causally to insecticide resistance but microsatellites, amplified fragment length polymorphisms (AFLPs) and some other molecular markers have been used to this end. Results for pyrethroid resistance in three mosquito species are typical, with one to three loci explaining most of the variation, but still leaving significant genetic variation to be explained by other, as yet unidentified, factors.^19^–^21^ Furthermore, intensive analysis of two major loci contributing to pyrethroid resistance in the malaria vector *Anopheles funestris* has recently found that each in fact includes at least two closely related P450 genes and each of these is over-expressed in resistant individuals.^22^,^23^

So how do we reconcile the different sorts of data? Is the genetic basis for field resistance actually more complex than the limited power of classical genetics applied to non-model insects can detect? Just how limiting are the genetic options available to confer insecticide resistance? Are there real differences between laboratory and field populations in respect of the genetic basis of resistance? Are there qualitative differences in the genetic complexity of resistances between insect orders or between polyphagous pests and more specialised feeders? Herein we present some important insights into these issues emerging from recent classical genetic, biochemical and genomic studies of OP and pyrethroid resistance in some highly polyphagous lepidopteran pests, specifically heliothines, particularly *Helicoverpa armigera*, and *Spodoptera*.

## 3 THE CLASSICAL GENETICS AND BIOCHEMISTRY OF THE LEPIDOPTERAN CASES

Numerous studies have now reported OP and pyrethroid resistances in various African, Asian and Australian populations of *H. armigera*, with some reports indicating resistance factors exceeding 100-fold for the OPs and 1000-fold for the pyrethroids.^10^,^24^ Bioassays with diagnostic synergists implicate predominantly esterase-and P450-based metabolic resistance mechanisms in both cases, although the relative importance of the two mechanisms varies between populations. Broadly similar findings have also emerged from several studies of resistances to the two chemistries in *Helicoverpa punctigera*, *Heliothis virescens*, *Spodpotera littoralis* and *Spodoptera litura* (with more limited data to the same effect also for *Spodoptera exigua* and *Spodoptera frugiperda*), although not always for both sets of chemistries and other mechanisms are also influential in some cases.^10^,^24^

Classical genetic analyses are difficult in these species and we have only found two relevant to our remit. The results of one back-crossing study on esterase-based pyrethroid resistance in an African *H. armigera* population were most consistent with the action of a single dominant autosomal gene, albeit minor effects of other genes or the combined effects of several closely linked genes could not be discounted by the data.^25^ The other study, on an *S. litura* strain which bioassay data indicated had P450-and some esterase-based pyrethroid resistance, found major effects of at least two dominant autosomal genes,^26^ although the precise number of genes could not be specified from the data and the caveats above about very limited power to detect genes of smaller effect or resolve the effects of closely linked genes again apply.

The biochemical phenotypes associated with the two resistance mechanisms in these species have almost always been elevations in the levels of esterase and P450 activities against artificial substrates in larval homogenates.^10^,^24^ Many of the individual studies reporting these associations could be criticised because of the limited number of strains involved, the different genetic backgrounds of the resistant and susceptible strains and, in some cases, the co-occurence of resistances to other chemistries in the resistant strains. However, the combined data across a large number of studies (approaching 30 for *H. armigera* alone) make a strong case. Moreover a few studies have shown a quantitative correlation between resistance levels and the esterase and/or P450 activities across strains, or over the course of laboratory selection for increased resistance, and subsequent relaxation of selection.^27^–^29^ Such patterns support the idea of a direct connection between the resistance and the biochemistry.

**Figure 1:**
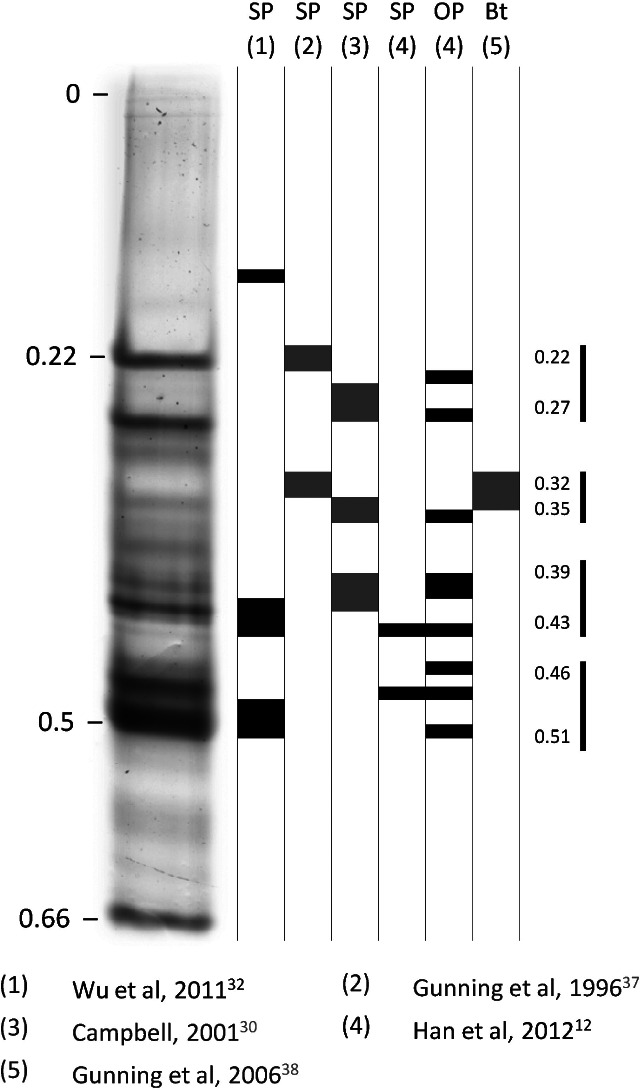
Isozyme regions associated with organophosphorus (OP), pyrethroid (SP) and *Bacillus thuringiensis* resistance in *Helicoverpa armigera* in five studies using comparable electrophoretic methods. The reference isozyme lane shown is from fifth instar larvae of the Australian GR strain and the schematic lanes shown for the five studies are coded black for Chinese and grey for Australian strains. The numbers in the margins refer to relative mobility (Rm) values.

The biochemical work on the esterases has gone a step further because of the facility of separating and staining individual esterase enzymes on native PAGE gels. The esterase isozyme profiles of the leipdopterans under discussion here are found to be considerably more complex than those of the dipteran and hemipteran precedents above. For example, Campbell^30^ found over 30 distinct isozymes in fourth instar *H. armigera* larvae alone, with several others also evident in other life stages of this species. Moreover greater staining intensities of several of the isozymes (6–9 in some cases) have been associated with OP or SP resistances in *H. armigera*,^12^,^31^,^32^ *H. punctigera*,^33^ *H. virescens*,^27^ *S. littoralis*,^34^ *S. litura*^35^ and *S. exigua*.^36^ Figure 1 shows that at least four regions of the esterase zymogram, each containing multiple isozymes, have been associated with OP and pyrethroid resistance in *H. armigera*.^12^,^30^,^32^,^37^,^38^

The biochemical mechanisms linking the more intensely staining isozymes to the resistances are not yet known but a mix of enhanced sequestration and metabolism of the insecticides is widely assumed to apply.^10^,^39^ In fact there is as yet little direct empirical evidence that the enzymes can actually bind or metabolise the insecticides. A couple of studies have shown esteratic cleavage of OPs and pyrethroids in whole larval extracts and there is also evidence for binding/inhibition of several of the relevant isozymes by the pesticides.^30^–^36^,^39^,^40^ Wu *et al*.^32^ also showed that an excised extract of one of the major isozymes associated with pyrethroid resistance in *H. armigera* was able to hydrolyse a pyrethroid, and purified esterases from other insects have been shown to have good activity for several pyrethroids, and much lower but possibly still protective activities against the activated, oxon forms of OPs.^41^ However, the outstanding need is for biochemical and physiological work testing directly for the sequestration and/or metabolic capabilities of the relevant heliothine and spodopoteran isozymes.

Intriguingly, some level of OP/pyrethroid cross-resistance which is synergised by esterase inhibitors has also been noted in some of the work on these species.^35^,^42^,^43^ It is not found in all such studies but, in some cases involving correlated responses in laboratory selection experiments, the evidence is quite strong and encompasses carbamates as well.^40^ Synergistic effects among the three classes of chemistry have been well documented and are commonly explained in terms of the sensitivity of the relevant detoxifying esterases to inhibition by OPs and carbamates, as per the sensitivity of the closely related acetylcholinesterase that is the primary target of OP and carbamate insecticides. It is assumed that some cross-resistance eventuates because over-expression of the relevant enzymes produces higher levels of sequestration/degradative activities for the different sets of chemistries. The overlap in isozyme bands associated with OP and pyrethroid resistances in *H. armigera* noted above is consistent with this idea.

## 4 INSIGHTS INTO THE LEPIDPOTERAN RESISTANCES FROM THE ‘OMICS’

**Figure 2:**
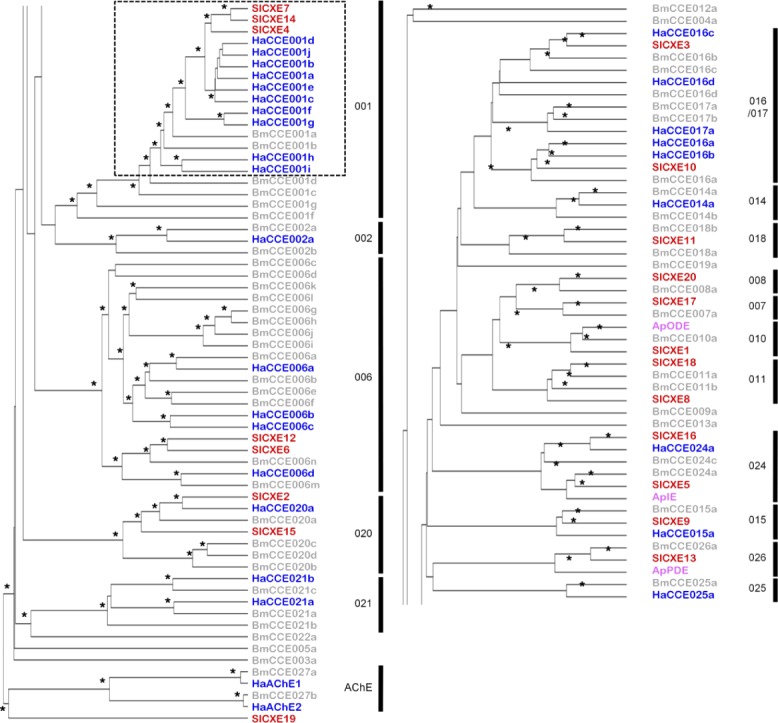
Phylogeny of the esterase genes recovered from the midgut transcriptome of *Helicoverpa armigera*,^32^,^45^ the antennal transcriptome of *Spodpotera littoralis*^46^,^47^ and the full genome sequence of *Bombyx mori*.^45^,^49^,^50^ Nomenclature is according to Teese *et al*.^45^ and Durand *et al*.^47^ The cladogram is based on an alignment of amino acid sequences trimmed as previously described (Claudianos *et al*.^13^) using default parameters of the sequence alignment and UGPMA tree construction algorithms of CLC Main Workbench 6 software, Version 6.5.5 (CLC-Bio). Asterisks indicate nodes supported by at least 50% of bootstrap replicates (*n* = 1000). Major clades are indicated by their number and a solid vertical bar and the section of clade 1 containing enzymes implicated in xenobiotic metabolism is boxed. The sequences BmCCE001e and BmCCE024b of Teese *et al*.^45^ subsequently identified as allelic variants of BmCCE001d and BmCCE024a, respectively (Tsubota and Shiotsuki^50^) were omitted from this analysis. The partial sequence BmCCE023a was excluded from the alignment, but the additional *H. armigera* clade 1 sequence HaCCE001j of Wu *et al*.,^32^ not available to Teese *et al*.,^45^ is included.

Projects to sequence the genomes of *H. armigera* and *S. littoralis* are under way but not yet completed. Meanwhile, however, tissue-specific transcriptome data are providing early insights into an unusual diversity of genes for P450s and esterases in heliothine and spodopteran pests. Sequencing of over 1600 paralogous midgut transcripts in *H. virescens* identified 20 different P450s and nine esterases,^44^ while a similar project on about 3000 unique midgut transcripts from *H. armigera* identified 40 paralogous esterases alone.^32^,^45^ Trancriptomes of adult antennae (which also appear to include many detoxification genes) likewise reveal large numbers of P450s and esterases, 37 and 30, respectively, in *S. littoralis*.^46^,^47^ Notably the CYP3 and −4 clans predominate among the antennal P450 transcripts, and the −4, −6, −9 and −12 clans among the midgut RNAs, with esterase clades CCE1, in particular, and −16/−17 found in the transcriptomes of both tissues. CYP-4, −6, −9 and −12 P450s are commonly associated with insecticide resistances in other organisms^48^ and the esterase clades −1 and −16/−17 have been variously associated with resistance in *H. armigera*.^12^ The phylogeny in Fig. 2 shows that the relevant section of esterase clade −1 indeed contains several more genes in *H. armigera* than in another lepidopteran which is highly host-specific, the silkworm *Bombyx mori*.^45^,^49^,^50^

Several studies have now compared the expression levels of various *CYP* genes between pyrethroid susceptible and resistant strains of *H. armigera* (Table 1).^51^–^57^ A total of 30 different *CYP* genes have been compared in this way, several in more than one study, and 12 have been found to be more highly expressed in the resistant material in at least one study. While some (e.g. *CYP6AE11*,-*6B7*,-*9A12*,-*9A14*,-*332A1,-337B1*) show this pattern in multiple studies, others (e.g.-*4G8*,-*4 L5*,-*4 L11*,-*4 M6*,-*4 M7*) show more study-/population-specific effects. Individual resistant populations are reported in which as many as five *CYP* genes have been found to be over-expressed.^57^ Given that only perhaps a third of the total complement of P450s in *H. armigera* have been included in any of these studies,^57^ these transcriptomic experiments suggest considerable scope for many *CYP* genes to be involved in pyrethroid resistance in this species, in either population-specific or more general ways.

**Table 1:** Relative expression levels of *CYP* genes in pyrethroid susceptible (S) and resistant (R) strains of *Helicoverpa armigera*

Authors and Reference	Country	Genes in which there is no difference	Genes in which R>S
Xiao-Ping and Hobbs^51^	Australia	6B2	
Pittendrigh *et al*^52^	Australia	4S1, 4S2, 4G9, 4G10, 4M4	4G8
		6B2	
		9A3	
		43L	
Ranasinghe and Hobbs^53^	Australia	6B6	6B7
Yang *et al*^54^	China	4G8	6B7
			9A12, 9A14
Wee *et al*^55^	Australia	?	4S1
			337B1
Zhang *et al*^56^	China		6B7
Brun-Barale *et al*^57^	Burkina-Faso, Spain	4G8, 4M6, 4M7, 4M10	4L5, 4L11
		6AB9, 6AE12, 6AE15, 6AE16, 6B6, 6B7	6AE11
		9A12, 9A16, 9A17, 9A23	9A14
		321A1, 337B1	332A1
	Benin, Burkina-Faso, Mali	4G8, 4L5, 4L11, 4M10	4M6, 4M7
		6AB9, 6AE12, 6AE15, 6AE16, 6B6, 6B7	6AE11
		9A14, 9A16, 9A17, 9A23	9A12
		321A	332A1, 337B1

R>S indicates that expression was significantly higher in the resistant than in the susceptible strain.

However, QTL mapping of pyrethroid resistance in *H. armigera* appears to tell a different story, with a single major locus found to account for most (∼80%) of the ∼50-fold P450-based metabolic resistance in an Australian population.^58^ This locus was found to encompass the *CYP337B1* gene which was over-expressed in some of the pyrethroid resistant strains above.^59^ *CYP337B1* is a hybrid of the closely linked *CYP337B2* and *CYP337B3* genes and produces an enzyme with an ability to metabolise pyrethroids that both the parental genes/enzymes lack.^59^ These findings are significant in several respects. On the one hand they caution against the widely made assumption that the greater P450 activities measured with artificial substrates reported in many of the biochemical studies outlined above are necessarily relevant to resistance. And they also caution against the assumption that resistance based on greater P450 activity would generally be achieved by over-expression of existing P450s rather than the creation of novel P450s (and there are other examples of resistance due to structural mutations creating enzymes with new activities, both among the P450s and among the esterases^5^). Nevertheless, we also note that many of the biochemical and transcriptomic studies above involved significantly higher P450-based resistance factors (e.g. ∼400-fold in Yang *et al*.^54^) and some did not involve any over-expression of *CYP337B1* (e.g. Brun-Barale *et al*.^57^). Thus *CYP337B1* only explains a proportion of the P450-based resistance so far described for this species.

There is no comparable QTL analysis of esterase-based resistances in the heliothines or *Spodoptera* but there are some intriguing data showing elevated expression of multiple esterases in resistant material, in this case involving both pyrethroids and OPs. Wu *et al*.^32^ and Han *et al*.^12^ studied two pyrethroid-and one OP-resistant Chinese strains of *H. armigera* which each produced isozyme profiles with between three and nine bands that were more intensely staining than in a control susceptible strain. Two of these bands were more intense in all three resistant strains but otherwise there were considerable differences between them. Proteomic analyses on the isozymes from one of the pyrethroid resistant strains and the OP resistant strain yielded matches to nine of the 30 esterase genes in the transcriptomic databases above, with six of the nine (*CCE001a*,-*1c,-1d*,-*1 g*,-*1i* and-*1j*) all being closely related to one another in a subclade that is considerably larger in *H. armigera* than in *B. mori* (8 cf 2 genes, respectively; see Fig. 2). The latter difference is also likely to be an underestimate because the *B. mori* figure is based on full genome sequence whereas the current transcriptome databases for *H. armigera* (and *S. littoralis*) may only contain about a third of its esterases.^45^,^49^,^50^ Native western analysis with a polyclonal antibody to the clade 1 esterases confirmed that the greater intensities of the corresponding isozyme bands in the resistant strain were due to greater amounts of the corresponding proteins. These esterase data thus bear out the findings from the P450 transcriptomics to date that a large number of often very closely related genes can be upregulated in resistant material, but that the specific genes upregulated can vary widely between strains.

## 5 CONCLUSIONS

A strong case emerges from a large number of studies for a direct link between OP and pyrethroid resistances and elevated levels of P450 and esterase activities in the heliothine and spodopteran species considered herein. Whilst some of the studies involve laboratory selection experiments, the husbandry and breeding issues with laboratory colonies of the species mean that the associations seen will generally reflect the effect of genes present in their wild/caught progenitors. The data already available also make a strong case for a direct link between the pyrethroid resistances in particular and elevated expression of several *CYP* and *CCE* genes, albeit some of the genes implicated in individual studies may well prove to be strain differences in genetic backgrounds rather than directly linked to the phenotype. It seems likely from studies such as those by Brun-Barale *et al*.^57^ and Han *et al*.^12^ that there are also genuine differences between populations in some of the specific genes that are upregulated in this way. Whilst the very limited number of classical genetic and QTL studies on the issue clearly point to some individual genes of major effect, it therefore seems likely that several members of the two families of detoxification genes could be involved in the resistances.

The transcriptome databases currently available for the heliothine and spodopteran species in question likewise suggest that there may be relatively large numbers of P450s and esterases that could provide options for the development of resistance if upregulated. These data include evidence for several more genes in *CYP* clans and *CCE* clades implicated in detoxification functions in *H. armigera* and *S. littoralis* than occur in, for example, *B. mori*.^45^,^49^,^50^

How then to resolve the paradox that relatively few major genes for resistance are identified in the classical genetic and QTL mapping but large numbers are implicated as upregulated in the biochemistry and transcriptomics?

One explanation may be that at least some of the latter are incidentally over-expressed as part of the upregulation of a more general stress response controlled by genes operating upstream in a regulatory cascade.^10^ Such P450s and esterases may have little or no degradative or binding activity against the insecticides or their metabolic products. Even assuming that several P450s and esterases do have physiologically important pyrethroid or OP degradative or sequestration activities, their titres may still be controlled by an upstream gene(s) in a regulatory cascade. In this case only the upstream regulatory gene(s) would contribute to the heritability of resistance and register in a classical genetic or QTL mapping experiment. The only case where a specific gene encoding a physiologically relevant (pyrethroid) detoxification activity is yet known to make a major contribution to the heritability of resistance is the *H. armigera CYP337B1* above.^59^ As noted, biochemical work is now required to test the metabolic activities of more of the upregulated P450s and esterases against the insecticides, and high density QTL mapping is required on more highly resistant strains to determine how the genes contributing to the heritability of resistances relate to the genes that actually encode the detoxifying functions. The application of new gene silencing technologies^60^ and improved abilities to express P450 genes *in vitro*^61^ should facilitate the biochemical work required.

As well as the possibility of mutation in an upstream regulatory gene, there is another major genetic reason why the number of genetic options available for the development of various resistances might be under-represented by even a quite powerful QTL analysis. This involves the common observation of large clusters of closely related esterase and P450 genes in the genome. Such clusters have been found in all the insect species for which full genome sequence data have so far been produced. For example, over half the *B. mori* esterase genes sit in clusters of up to seven tandemly arranged members,^49^ while a blowfly esterase implicated in major gene control of OP resistance sits in a cluster of at least eight members.^62^ Tightly linked clusters such as these generally behave as single loci in QTL experiments^22^ (and even genome-wide association studies^63^), even though it is quite possible that several of their component genes could be independently upregulated in resistant strains. Similarly, two such tightly linked genes could contribute to resistance to different chemistries but co-segregate in both genetic mapping and laboratory selection experiments, giving an appearance of a shared cross-resistance phenotype.

Additionally we suggest that, while several esterases and P450s may indeed be selected for by OP or pyrethroid exposure, their relative contributions to resistance might be expected to vary substantially, depending on their biochemistry and physiology. A small number may exert major effects while polygenic effects result from smaller contributions from several others. This is clearly the case in the best characterised mosquito case (the pyrethroid resistance in *A. funestris* outlined above).^22^,^23^ The findings from the very extensive insecticide toxicology literature for mammals are also worth noting in this respect; the general findings are that the metabolism of most insecticides involves several enzymes which vary in their contributions to overall detoxification.^64^

Finally, we note an emerging and still contentious literature reporting an association in *H. armigera* between midgut esterase activities and resistance to the Cry1Ac toxin of the biopesticide *Bacillus thuringiensis* which is now widely used in transgenic strategies for conferring endogenous insect protection on crops.^38^,^45^,^65^,^66^ The esterases involved migrate to the same zymogram region as one of those implicated in OP and pyrethroid resistance (see Figure 1). The mechanism underlying the association with Cry1Ac resistance is unclear but its existence is supported by the finding of a similar association in the diamondback moth *Plutella xylostella*.^67^ Further work is required to determine the generality and the basis of the association, and how it impacts on the associations of the esterases with the OP and pyrethroid resistances outlined herein.

## References

[1] Roush RT, McKenzie JA (1987). Ecological genetics of insecticide and acaricide resistance. Ann Rev Entomol.

[2] Russell RJ, Claudianos C, Campbell PM, Horne I, Sutherland TD, Oakeshott JG (2004). Two major classes of target site insensitivity mutations confer resistance to organophosphate and carbamate insecticides. Pestic Biochem Physiol.

[3] Lima EP, Paiva MHS, de Araújo AP, da Silva EVG, da Silva UM, de Oliveira LN (2011). Insecticide resistance in *Aedes aegypti* populations from Ceará, Brazil. Parasites Vectors.

[4] Perry T, Batterham P, Daborn PJ (2011). The biology of insecticidal activity and resistance. Insect Biochem Mol Biol.

[5] Hartley CJ, Newcomb RD, Russell RJ, Yong CG, Stevens JR, Yeates DK (2006). Amplification of DNA from preserved specimens shows blowflies were preadapted for the rapid evolution of insecticide resistance. Proc Natl Acad Sci U S A.

[6] Wang Q, Li M, Pan J, Di M, Liu Q, Meng F (2012). Diversity and frequencies of genetic mutations involved in insecticide resistance in field populations of the house fly (*Musca domestica* L.) from China. Pestic Biochem Physiol.

[7] Van Leeuwen T, Vontas J, Tsagkarakou A, Dermauw W, Tirry L (2010). Acaricide resistance mechanisms in the two-spotted mite *Tetranychus urticae* and other important Acari: A review. Insect Biochem Mol Biol.

[8] Scott JG, Kasai S (2004). Evolutionary plasticity of monooxygenase-mediated resistance. Pestic Biochem Physiol.

[9] Li X, Schuler MA, Berenbaum MR (2007). Molecular mechanisms of metabolic resistance to synthetic and natural xenobiotics. Ann Rev Entomol.

[10] Farnsworth CA, Teese MG, Yuan G, Li Y, Scott C, Zhang X (2010). Esterase-based metabolic resistance to insecticides in heliothine and spodopteran pests. J Pestic Sci.

[11] Heckel D, Goldsmith MR, Marec F (2012). Molecular genetics of insecticide resistance in Lepidoptera. Molecular Biology and Genetics of the Lepidoptera.

[12] Han Y, Wu S, Li Y, Liu J-W, Campbell PM, Farnsworth C (2012). Proteomic and molecular analyses of esterases associated with monocrotophos resistance in *Helicoverpa armigera*. Pestic Biochem Physiol.

[13] Claudianos C, Ranson H, Johnson RM, Biswas S, Schuler MA, Berenbaum MR (2006). A deficit of detoxification enzymes: Pesticide sensitivity and environmental response in the honeybee. Insect Mol Biol.

[14] Oakeshott JG, Johnson RM, Berenbaum MR, Ranson H, Cristino RS, Claudianos C (2010). Metabolic enzymes associated with xenobiotic and chemosensory responses in *Nasonia vitripennis*. Insect Mol Biol.

[15] Lee SH, Kang JS, Min JS, Yoon KS, Strycharz JP, Johnson R (2010). Decreased detoxification genes and genome size make the human body louse an efficient model to study xenobiotic metabolism. Insect Mol Biol.

[16] Strode C, de Melo-Santos M, Magalhães T, Araújo A, Ayres C (2012). Expression profile of genes during resistance reversal in a temephos selected strain of the Dengue vector, *Aedes aegypti*. PLoS One.

[17] Saavedra-Rodriguez K, Suarez AF, Salas IF, Strode C, Ranson H, Hemingway J (2012). Transcription of detoxification genes after permethrin selection in the mosquito *Aedes aegypti*. Insect Mol Biol.

[18] Müller P, Warr E, Stevenson B, Pignatelli PM, Morgan JC, Steven A (2008). Field-caught permethrin-resistant *Anopheles gambiae* overexpress CYP6P3, a P450 that metabolises pyrethroids. PLoS Genet.

[19] Ranson H, Paton MG, Jensen B, McCarroll L, Vaughan A, Hogan JR (2004). Genetic mapping of genes conferring permethrin resistance in the malaria vector, *Anopheles gambiae*. Insect Mol Biol.

[20] Wondji CS, Morgan J, Coetzee M, Hunt RH, Steen K, Black WC (2007). Mapping a quantitative trait locus (QTL) conferring pyrethroid resistance in the Afrcian malaria vector *Anopheles funestus*. BMC Genomics.

[21] Saavedra-Rodriguez K, Strode C, Suarez AF, Salas IF, Ranson H, Hemingway J (2008). Quantitative trait mapping of genome regions controlling permethrin resistance in the mosquito *Aedes aegypti*. Genetics.

[22] Irving H, Riveron JM, Ibrahim SS, Lobo NF, Wondji CS (2012). Positional cloning of rp2 QTL associates the P450 genes *CYP6Z1**CYP6Z3* and *CYP6M7* with pyrethroid resistance in the malaria vector *Anopheles funestris*. Heredity.

[23] Riveron JM, Irving H, Ndula M, Barnes KG, Ibrahim SS, Paine MJI (2013). Directionally selected cytochrome P450 alleles are driving the spread of pyrethroid resistance in the major malaria vector *Anopheles funestris*. Proc Natl Acad Sci U S A.

[24] McCaffery AR (1998). Resistance to insecticides in heliothine Lepidoptera: A global view. Philos Trans R Soc London Ser B.

[25] Achaleke J, Brévault T (2010). Inheritance and stability of pyrethroid resistance in the cotton bollworm *Helicoverpa armigera* (Lepidoptera: Noctuidae) in central Africa. Pest Manag Sci.

[26] Ahmad M, Sayyed AH, Crickmore N, Saleem MA (2007). Inheritance of resistance to deltamethrin in a field population of *Spodoptera litura* (Lepidoptera: Noctuidae) in Pakistan. Pest Manag Sci.

[27] Harold JA, Ottea JA (2000). Characterisation of esterases associated with profenofos resistance in the tobacco budworm, *Heliothis virescens*. Arch Insect Biochem Physiol.

[28] Zheng Y-P, Yang Y-H, Wu Y-D (2008). Metabolic resistance mechanisms in a Phoxim-resistant strain of *Helicoverpa armigera*. Chin J Pestic Sci.

[29] Xiao P, He J, Liu Y-J, Qiu X-C, Jiao Y-Y (2009). The relationship of resistance to lambda-cyhalothrin with detoxification enzyme activity in *Spodoptera litura* (Fabricius) (Lepidoptera: Noctuidae). Acta Entomol Sin.

[30] Campbell BE (2001).

[31] Gunning RV, Moores GD, Devonshire AL (1999). Esterase inhibitors synergise the toxicity of pyrethroids in Australian *Helicoverpa armigera* (Hübner) (Lepidoptera: Noctuidae). Pestic Biochem Physiol.

[32] Wu S, Yang Y, Yuan G, Campbell PM, Teese MG, Russell RJ (2011). Overexpressed esterases in a fenvalerate resistant strain of the cotton bollworm, *Helicoverpa armigera*. Insect Biochem Mol Biol.

[33] Gunning RV, Moores GD, Devonshire AL (1997). Esterases and fenvalerate resistance in a field population of *Helicoverpa punctigera* (Lepidoptera: Noctuidae) in Australia. Pestic Biochem Physiol.

[34] El-Guindy MA, Saleh WS, El-Refai AA, Abou-Donia SA (1985). The role of haemolymph esterases as protectants against intoxication by fenitrothion in the cotton leafworm *Spodoptera littoralis* (Boisd.). Bull Entomol Soc Egypt Econ Ser.

[35] Cho JR, Kim YJ, Kim JJ, Kim HS, Yoo JK, Lee JO (1999). Electrophoretic pattern of larval esterases in field and laboratory-selected strains of the tobacco cutworm *Spodoptera litura* (Fabricius). J Asia–Pacific Entomol.

[36] Kim YG, Lee JI, Kang SY, Han SC (1997). Variation in insecticide susceptibilities of the beet armyworm, *Spodoptera exigua* (Hübner): esterase and acetylcholinesterase activities. Korean J Appl Entomol.

[37] Gunning RV, Moores GD, Devonshire AL (1996). Esterases and esfenvalerate resistance in Australian *Helicoverpa armigera* (Hübner) Lepidoptera: Noctuidae. Pest Biochem Physiol.

[38] Gunning RV, Nicholson IC, Kemp FC, Borzatta V, Cottage E, Field LM (2006). Piperonyl butoxide restores the efficacy of *Bacillus thuringiensis* toxin in transgenic cotton against resistant *Helicoverpa armigera*. Biopest Int.

[39] Young SJ, Gunning RV, Moores GD (2005). The effect of piperonyl butoxide on pyrethroid resistance-associated esterases in *Helicoverpa armigera* (Hübner) (Lepidoptera: Noctuidae). Pest Manag Sci.

[40] Huang H, Ottea JA (2004). Development of pyrethroid substrates for esterases associated with pyrethroid resistance in the tobacco budworm, *Heliothis virescens* (F.). J Agric Food Chem.

[41] Coppin CW, Jackson CJ, Sutherland T, Hart PJ, Devonshire AL, Russell RJ (2012). Testing the evolvability of an insect carboxylesterase for the detoxification of synthetic insecticides. Insect Biochem Mol Biol.

[42] Zhao G, Rose RL, Hodgson E, Roe RM (1996). Biochemical mechanisms and diagnostic microassays for pyrethroid, carbamate and organophosphate insecticide resistance/cross-resistance in the tobacco budworm, *Heliothis virescens*. Pestic Biochem Physiol.

[43] Huang S, Han Z (2007). Mechanisms for multiple resistances in field populations of common cutworm *Spodoptera litura* (Fabricius) in China. Pestic Biochem Physiol.

[44] Zhu YC, Guo Z, Chen M-S, Zhu KY, Liu XF, Scheffler B (2011). Major putative pesticide receptors, detoxification enzymes, and transcriptional profile of the midgut of the tobacco budworm, *Heliothis virescens* (Lepidoptera: Noctuidae). J Invert Pathol.

[45] Teese MG, Campbell PM, Scott C, Gordon KHJ, Southon A, Robin C (2010). Gene identification and proteomic analysis of the esterases of the cotton bollworm, *Helicoverpa armigera*. Insect Biochem Mol Biol.

[46] Pottier M-A, Bozzolan F, Chertemps T, Jacquin-Joly E, Lalouette L, Siaussat D (2012). Cytochrome P450s and cytochrome P450 reductase in the olfactory organ of the cotton leafworm *Spodoptera littoralis*. Insect Mol Biol.

[47] Durand N, Chertemps T, Maïbèche-Coisne M (2012). Antennal carboxylesterases in a moth, structural and functional diversity. Commun Integr Biol.

[48] Feyereisen R (2006). Evolution of insect P450. Biochem Soc Trans.

[49] Yu Q-Y, Lu C, Li W-L, Xiang Z-H, Zhang Z (2009). Annotation and expression of carboxylesterases in the silkworm, *Bombyx mori*. BMC Genomics.

[50] Tsubota T, Shiotsuki T (2010). Genomic analysis of carboxyl/cholinesterase genes in the silkworm *Bombyx mori*. BMC Genomics.

[51] Xiao-Ping W, Hobbs AA (1995). Isolation and sequence analysis of a cDNA clone for a pyrethroid inducible cytochrome P450 from *Helicoverpa armigera*. Insect Biochem Mol Biol.

[52] Pittendrigh B, Aronstein K, Zinkovsky E, Andreev O, Campbell B, Daly J (1997). Cytochrome P450 genes from *Helicoverpa armigera*: expression in a pyrethroid-susceptible and-resistant strain. Insect Biochem Mol Biol.

[53] Ranasinghe C, Hobbs AA (1998). Isolation and characterisation of two cytochrome P450 cDNA clones for CYP6B6 and CYP6B7 from *Helicoverpa armigera* (Hübner): Possible involvement of CYP6B7 in pyrethroid resistance. Insect Biochem Mol Biol.

[54] Yang Y, Chen S, Wu S, Yue Y, Wu Y (2006). Constitutive overexpression of multiple P450 genes associated with pyrethroid resistance in *Helicoverpa armigera*. J Econ Entomol.

[55] Wee CW, Lee SF, Robin C, Heckel DG (2008). Identification of candidate genes for fenvalerate resistance in *Helicoverpa armigera* using cDNA-AFLP. Insect Mol Biol.

[56] Zhang H, Tang T, Cheng Y, Shui R, Zhang W, Qiu L (2010). Cloning and expression of cytochrome P450 *CYP6B7* in fenvalerate-resistant and susceptible *Helicoverpa armigera* (Hübner) from China. J Appl Entomol.

[57] Brun-Barale A, Héma O, Martin T, Suraporn S, Audant P, Sezutsu H (2010). Multiple P450 genes overexpressed in deltamethrin-resistant strains of *Helicoverpa armigera*. Pest Manag Sci.

[58] Grubor VD, Heckel DG (2007). Evaluation of the role of CYP6B cytochrome P450s in pyrethroid resistant Australian *Helicoverpa armigera*. Insect Mol Biol.

[59] Joußen N, Agnolet S, Lorenz S, Schöne SE, Ellinger R, Schneider B (2012). Resistance of Australian *Helicoverpa armigera* to fenvalerate is due to the chimeric P450 enzyme CYP337B3. Proc Natl Acad Sci U S A.

[60] Mao YB, Cai WJ, Wang JW, Hong GJ, Tao XY, Wang LJ (2007). Silencing a cotton bollworm P450 monoxygenase gene by plant-mediated RNAi impairs larval tolerance of gossypol. Nat Biotechnol.

[61] Cheesman MJ, Traylor MJ, Hilton ME, Richards KE, Taylor MC, Gillam EMJ (2013). Soluble and membrane-bound *Drosophila melanogaster* CYP6G1 expressed in *E. coli*: purification, activity and binding properties towards multiple pesticides. Insect Biochem Mol Biol.

[62] Newcomb RD, East PD, Russell RJ, Oakeshott JG (1996). Isolation of α-cluster esterases associated with organophosphate resistance in *Lucilia cuprina*. Insect Mol Biol.

[63] Rose CJ, Chapman JR, Marshall SDG, Lee SF, Batterham P, Ross HA (2011). Selective sweeps at the organophosphorus resistance locus, *Rop-1*, have affected variation across and beyond the α-esterase gene cluster in the Australian sheep blowfly, *Lucilia cuprina*. Mol Biol Evol.

[64] Marrs T (2012). Mammalian Toxicology of Insecticides.

[65] Gunning RV, Dang HT, Kemp FC, Nicholson IC, Moores GD (2005). New resistance mechanism in *Helicoverpa armigera* threatens transgenic crops expressing *Bacillus thuringinesis* Cry1Ac toxin. Appl Environ Microbiol.

[66] Alvi AHK, Sayyed AH, Naeem M, Ali M (2012). Field evolved resistance in *Helicoverpa armigera* (Lepidoptera: Noctuidae) to *Bacillus thuringiensis* toxin Cry1Ac in Pakistan. PloS One.

[67] Sayyed AH, Moores G, Crickmore N, Wright DJ (2008). Cross-resistance between a *Bacillus thuringiensis* Cry toxin and non-Bt insecticides in the diamondback moth. Pest Manag Sci.

